# Toward personalized medicine for pharmacological interventions in neonates using vital signs

**DOI:** 10.1002/pne2.12065

**Published:** 2021-11-22

**Authors:** Caroline Hartley

**Affiliations:** ^1^ Department of Paediatrics University of Oxford Oxford UK

**Keywords:** analgesics, neonate, pharmacodynamics, preterm, vital signs

## Abstract

Vital signs, such as heart rate and oxygen saturation, are continuously monitored for infants in neonatal care units. Pharmacological interventions can alter an infant's vital signs, either as an intended effect or as a side effect, and consequently could provide an approach to explore the wide variability in pharmacodynamics across infants and could be used to develop models to predict outcome (efficacy or adverse effects) in an individual infant. This will enable doses to be tailored according to the individual, shifting the balance toward efficacy and away from the adverse effects of a drug. Pharmacological analgesics are frequently not given in part due to the risk of adverse effects, yet this exposes infants to the short‐ and long‐term effects of painful procedures. Personalized analgesic dosing will be an important step forward in providing safer effective pain relief in infants. The aim of this paper was to describe a framework to develop predictive models of drug outcome from analysis of vital signs data, focusing on analgesics as a representative example. This framework investigates changes in vital signs in response to the analgesic (prior to the painful procedure) and proposes using machine learning to examine if these changes are predictive of outcome—either efficacy (with pain response measured using a multimodal approach, as changes in vital signs alone have limited sensitivity and specificity) or adverse effects. The framework could be applied to both preterm and term infants in neonatal care units, as well as older children. Sharing vital signs data are proposed as a means to achieve this aim and bring personalized medicine rapidly to the forefront in neonatology.

## 1 INTRODUCTION

Hospitalized infants frequently require drugs as part of their essential medical care^1^ but many are used off‐label and administered according to standardized dosing regimens, usually based on the weight or age of the infant.^2^, ^3^, ^4^ When providing a pharmacological intervention, clinicians must try to ensure a balance between efficacy and the adverse effects of a drug.^5^ However, there is wide variation in individual differences in both pharmacokinetics and pharmacodynamics, making drug administration a complex issue.^6^, ^7^ Pharmacokinetic models have been developed for commonly used analgesics, including paracetamol and morphine, with relationships between the weight of the infant and drug clearance identified.^8^, ^9^ Morphine clearance also changes drastically with postnatal age, with a recommended reduction of 50% of the dose in neonates younger than 10 days compared with older infants.^10^ Nevertheless, data are lacking from the youngest preterm infants and there is still large inter‐infant variation in drug action not explained by these models.^9^

Drug regimens that are tailored to an individual infant are needed to ensure efficacy is attained with limited side effects. Here I propose that analysis of vital signs data, which are routinely monitored in neonatal units, will provide an important avenue through which personalized pharmacological dosing regimens can be developed. I discuss approaches through which this could be achieved and a framework for analysis, and I suggest that by sharing data we can ensure that vital signs – an easily obtainable, but currently drastically underused, resource can enable tailored dosing regimens in neonates. Throughout this article the term vital signs will be used broadly to refer to measures of the body's vital functions, including heart rate and respiratory rate, as well as other routinely monitored measures of physiology and the term vital signs data will be used to also refer to the signals used to derive these measures (eg, ECG – electrocardiography). Analysis of vital signs should include more complex measures that can be derived from these signals such as heart rate variability. These measures could be used to develop personalized drug dosing—whereby dosing is adjusted, within the limits of clinical guidelines, for an individual infant to achieve greater efficacy and limit adverse effects. Importantly, as discussed further below, by using detailed analysis of the infant's own vital signs the proposed models should improve upon current methods where dosing is usually adjusted based on extreme events and snapshot views of vital signs^11^ and does not account for more subtle changes in vital signs related to the drug or possible nonlinear relationships with outcome.

This review will focus on the use of analgesics as an important example. Hospitalized infants require numerous painful procedures as part of their standard care, yet analgesics are often not given, in part owing to concerns around adverse effects.^12^, ^13^ Tailoring doses of analgesics to individual infants could enable safer and more effective medication administration. This is critical given both the short‐ and long‐term effects of pain in infants.^14^ For individualized dosing of an analgesic, two factors should be considered as follows: (a) individual differences in pain sensitivity/the response to painful procedures, which may mean a given infant requires different levels of analgesia, and (b) individual infant differences in the pharmacokinetics and pharmacodynamics of the analgesic. This review will focus on an aspect of the latter, suggesting assessing individual differences in changes in vital signs, as one component of pharmacodynamics, to determine whether these changes are predictive of outcome; however, we will first briefly consider the former.

## 2 INDIVIDUAL DIFFERENCES IN PAIN SENSITIVITY

Infants, like adults, have wide individual variability in the way they respond to a painful stimulus, which may be modulated by numerous factors, including stress, sex, and mode of delivery.^15^, ^16^, ^17^, ^18^ Furthermore, due to rapid development across the neonatal period, there is large variability in the way in which a single infant responds to painful procedures across their stay in neonatal care.^19^, ^20^, ^21^, ^22^, ^23^ Predicting individual differences in pain sensitivity and titrating the dose of an analgesic accordingly will be an important step toward individualized analgesic treatment. This could be achieved, for example, by using low level experimental noxious stimuli prior to a painful clinical procedure to gage an individual infant's nociceptive sensitivity.^24^ Previous responses to painful procedures could also be used to predict how sensitive an individual might be to a future painful procedure and, therefore, useful for informing future analgesic requirements. Moreover, recent work has demonstrated that resting state brain activity recorded using MRI can predict an individual infant's brain activity response to noxious stimuli,^25^ suggesting that baseline measures could be used to predict an individual infant's response to a painful procedure and consequently their analgesic requirements. While it is not currently clinically feasible to use measures of brain activity to predict an individual's response to a painful procedure, the development of clinically useable tools to assess both noxious‐evoked and baseline brain activity are an import avenue for improving the utility of these techniques.^26^ Moreover, further research in this area should ascertain whether and how brain activity measures relate to clinical pain assessment tools and investigate whether clinical pain assessment tools could predict responses to future painful procedures and consequentially analgesic requirements.

## 3 INDIVIDUAL INFANT DIFFERENCES IN PHARMACOKINETICS AND PHARMACODYNAMICS

Pharmacodynamics and pharmacokinetics of an analgesic vary across individuals. Factors such as receptor expression, drug absorption, and clearance, and body composition all change rapidly across the neonatal period, and it is essential to take postmenstrual and postnatal age into account with drug dosing,^9^ as well as other keys factors such as comorbidities and concomitant medication. Population‐derived pharmacokinetic models exist for paracetamol and morphine use in neonates,^9^ however, even with using these models, there is wide inter‐individual variation in pharmacokinetics and pharmacodynamics and it is essential that we better assess individual differences if we are to optimize analgesic dosing in infants. In the remainder of this article, I will concentrate on how we could assess individual differences in changes in vital signs data following drug administration and use these data to build models predicting individual dose requirements. While changes in vital signs are only one limited component of pharmacodynamics, many drugs used in neonatal care alter infant's vital signs, either as the intended effect or as a side effect, and vital signs data are routinely collected in neonatal units providing an ideal opportunity to explore inter‐individual variation. It is plausible that any changes in vital signs in an individual following analgesic administration relate (possibly through complex nonlinear relationships) to the efficacy and adverse effects of the analgesic in that individual, and so investigating models of these relationships warrants further investigation.

Recently, Vinks and colleagues developed an electronic health record‐integrated, decision support platform for individualized precision dosing of morphine in the management of neonatal pain.^27^ A pharmacokinetic model is integrated with feedback from the individual through measured morphine concentrations to give an individual infant time profile for concentration levels. This is reported in a user‐friendly interface simultaneously with pain scores for the infant to aid clinical decision making and is a significant step toward assessing individual dosing requirements. The modelling proposed here, with real‐time assessment of vital signs, could enable further tailoring of a dose to an individual and should be combined with the substantial knowledge of infant drug dosing which already exists.

## 4 VITAL SIGNS—AN UNDERUTILIZED RESOURCE?

Vital signs are monitored continuously in infants in neonatal care and are sampled up to hundreds of times a second for traces such as the ECG, impedance pneumograph and photoplethysmograph, and around once a second for derived measures such as heart rate, respiratory rate, and oxygen saturation.^28^, ^29^ While some neonatal units are now storing electronic records of these data, in many hospitals it is not saved, and instead nursing observations of events such as episodes of oxygen desaturation, bradycardia and tachycardia, are recorded on clinical charts allowing for review of trends in an infant's data. These records will document the most severe episodes of physiological instability but provide only an intermittent and somewhat subjective view of the infant's physiology,^30^ will miss more subtle changes in an infant's vital signs that may be caused by pharmacological interventions, may be unreliable due to known problems in vital signs monitoring (particularly with regard to missing episodes of apnea due to cardiac interference on respiratory recordings),^31^, ^32^, ^33^, ^34^ and do not allow for more complex analysis that may reveal important predictive features within the data. For example, we have recently developed an algorithm to better identify inter‐breath intervals and episodes of apnea from the chest electrical impedance pneumograph; using this we found that 88% of apneas (defined as pauses in breathing of at least 20 seconds) identified using our method were not recorded on clinical notes and demonstrated a significant increase in pauses in breathing of at least 10 seconds following retinopathy of prematurity screening, which is not reflected in clinical notes or in changes in the respiratory rate recorded directly on the monitor.^34^ Similarly, Vergales et al found that more than 70% of apneas (of duration of at least 30 seconds and accompanied by bradycardia and desaturation) identified using an algorithm developed by their group were missed in clinical notes.^33^ This highlights the importance of using vital signs recordings directly rather than clinical records and the improvements that could be made in personalized drug dosing through detailed analysis of vital signs compared with clinical intermittent observations of the data.

Previous work has demonstrated the great potential of more detailed analysis of vital signs in providing early prediction of pathology such as sepsis, necrotizing enterocolitis (NEC) and bronchopulmonary dysplasia (BPD).^35^ These models have often combined direct measures of physiology, such as heart rate, with more complex derived measures such as heart rate variability^36^ or area‐under‐the‐curve of thresholded oxygen saturations.^37^ In the field of pharmacology, recent analysis of electronically captured vital signs has begun to demonstrate the potential vital signs data have for better informing drug provision and dosing in neonatal units. Poppe et al^38^ retrospectively identified responders and nonresponders to doxapram therapy (a respiratory stimulant sometimes used as an adjunct to caffeine therapy for the treatment of apnea of prematurity, though not currently recommended for routine use due to limited evidence of efficacy and safety),^39^ and those infants who were overexposed and risked unnecessary adverse effects. Interestingly, their case series also presented infants who may have unnecessarily started doxapram despite clinical review of observational charts at the time suggesting its indication, highlighting the disadvantage of intermittent review of vital signs. In a more detailed analysis of vital signs data from 61 infants, the same authors demonstrated that the ratio of oxygen saturation (SpO_2_)/fraction of inspired oxygen (FiO_2_), corrected for postmenstrual age and mechanical ventilation requirements, before the start of therapy discriminated well between infants where doxapram therapy failed (defined as subsequent intubation or death) and succeeded,^11^ indicating that predictive modelling could be a useful tool for individualized pharmacotherapy.

The idea presented here to use vital signs to assess drug effects in itself is not new—care providers will frequently titrate drugs for an individual based on changes in the infant's vital signs (as well as other features). Here, I am proposing a similar strategy, of real‐time modifications in drug dosing based on vital signs changes. However, changes in drug dosing currently in place are often based on snapshot impressions of the vital signs and the more severe adverse events. More subtle changes in vital signs or changes in more complex parameters such as heart rate variability will be missed. To fully utilize the potential of vital signs for tailored dosing regimens I believe we need to use a data driven approach, ultimately providing a tool that clinicians can use to aid with drug dosing.

## 5 HOW COULD VITAL SIGNS BE A USEFUL TOOL FOR ASSESSING INDIVIDUAL DIFFERENCES IN ANALGESIC ACTION?

I propose that detailed analysis of changes in vital signs following analgesic administration will provide pharmacodynamic biomarkers (ie, defined characteristics measured as an indicator of a biological response to the drug—for definitions of biomarkers, including pharmacodynamic biomarker, see the FDA‐NIH Biomarker Working Group BEST Resource^40^) in an individual, which may relate to efficacy or adverse effects and so could enable tailored analgesic dosing in infants. For example, while morphine can cause respiratory depression in some individuals, smaller physiological side effects such as a change in heart rate can be observed even in infants without respiratory depression, and the degree of this change in heart rate varies between infants given the same dose of morphine.^41^ Early changes in vital signs (in the minutes after a drug has been given, and before a painful procedure is performed) will be a useful indication of the action of the analgesic in that individual. If the changes in vital signs are low or absent, then this may be an indication that the infant requires a higher dose to have analgesic efficacy. Conversely, if the changes in vital signs are marked then this is likely an indication that the infant should not be given any further doses (unless the benefit is determined to outweigh the risk of side effects and the infant can be appropriately monitored and side effects managed^5^). By investigating how changes in vital signs relate to measures of the analgesic efficacy and adverse effects we may be able to titrate the dose for an individual prior to the painful procedure to improve pain management.

In addition, the trajectory of changes in vital signs in an individual could be used to optimally time the painful procedure to the peak effect of the analgesic. Many known factors affect the timing of peak effect of a drug, for example, the route of administration, the loading dose, the site of action and distribution of the drug. Pharmacokinetic models could be integrated to compare the time of peak effect with the estimated trajectory following the vital signs data. Investigating the relationship between initial drug action on the vital signs and outcome (response to a painful procedure or adverse effects) in a large cohort of infants will enable vital signs features to be identified that are predictive of outcome, and translation of such a model to the clinical setting could aid decision making with regard to modifications in drug dosing for an individual.

It is important here that we make the distinction between (a) the effect of the analgesic on vital signs prior to the painful procedure being performed, and (b) the combined effect of the painful procedure and the analgesic on the vital signs. Here, we consider the initial (before the painful procedure) effect of the analgesic on vital signs and compare this with the infant's response to the painful procedure (ie, an assessment of analgesic efficacy) in order to build a model to predict this relationship in future infants. Crucially though, the infant's pain response should be assessed using a multimodal approach and not limited to the vital signs’ response to the painful procedure. Although initial changes in vital signs prior to the painful procedure may be related to changes in vital signs in response to the painful procedure, tailoring the dose of an analgesic after the painful procedure for which it is given has no utility—these methods are only useful if we can adjust the dose of a drug accordingly before the painful procedure. Moreover, while changes in vital signs such as heart rate and oxygen saturation are frequently incorporated into pain assessment tools, these measures may lack sensitivity and specificity.^42^ Nociceptive input elicits responses at many different levels of the nervous system and a multimodal approach, including assessment of changes in vital signs, has been advocated to assess analgesic efficacy.^23^, ^43^, ^44^ The specific measure(s) that should be used to assess analgesic efficacy are still much debated and a discussion of this is beyond the scope of this article.^4^

## 6 USING VITAL SIGNS TO TAILOR ANALGESIC DOSING REGIMENS FOR INDIVIDUAL INFANTS—A FRAMEWORK FOR ANALYSIS

Here, I set out a framework to develop tools for personalized drug dosing in infants using vital signs. This framework makes the assumption that an analgesic is being prescribed for a particular procedure and so we can measure vital signs before and after (the start of) analgesic administration; for cases where we cannot measure vital signs prior to drug administration an alternative framework would be necessary, which will not be discussed here. Background vital signs (prior to drug administration) are essential to identify change in each individual's vital signs and will allow the model to account for the myriad of other factors, which could affect vital signs such as the age of the infant, mode of respiratory support, concomitant medication, and comorbidities.

**FIGURE 1:**
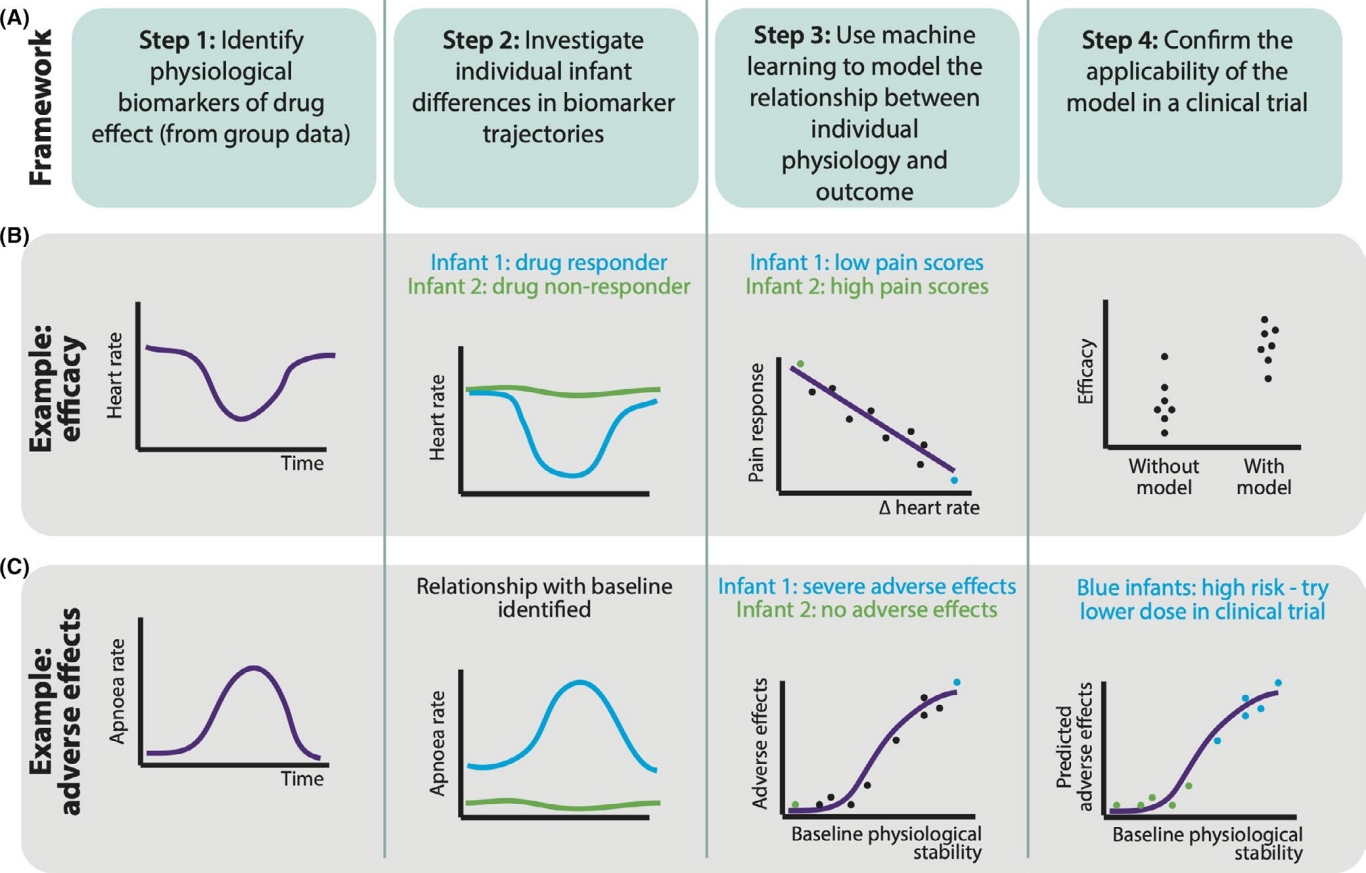
Framework for the analysis of vital signs data to develop models for individualized analgesic dosing regimens. The framework for analysis is presented (A) with two hypothetical examples (B,C), which are described further in the main text

The first step in the framework (Figure 1A) is to use recordings of vital signs data to identify biomarkers of the effect of a given analgesic (ie, a characteristic(s), such as a change in heart rate, heart rate variability, respiratory rate variability, and so on that can be used to measure whether an infant has responded to the analgesic). Importantly, this is not the same as identifying adverse effects from vital signs; smaller changes in vital signs that are not clinically significant will be useful indicators of analgesic action. To identify potential biomarkers, vital signs data should be collected for several hours before and after analgesic administration, and changes in vital signs investigated by first averaging the data from all infants in an exploratory data set. Changes in more complex derived measures (such as heart rate variability or respiratory rate variability) should be explored. Importantly, as discussed, these measures should be assessed prior to the painful procedure so that any changes identified are specific to the effect of the analgesic alone.

Having identified possible biomarkers of drug action by averaging across all infants, we must then investigate if these measures vary across individuals (Step 2, Figure 1A) and are predictive of outcome—for example, analgesic efficacy or occurrence of adverse events (Step 3 Figure 1A). For example, we might find that heart rate variability drops immediately following drug administration in some infants and not in others, and that this is related to the infants’ responses to a painful procedure performed after analgesic administration (and assessed using a multimodal approach^44^). Crucially here the drop in heart rate variability is in response to drug administration, not the painful procedure. Heart rate variability is, therefore, not a measure of analgesic efficacy, it is a marker of the effect of the analgesic on the infant's vital signs, which may be related to the analgesic efficacy in an individual.

To determine whether the changes in vital signs (and derived measures) that we observe in individual infants following drug administration are predictive of outcome and to inform us of the generalizability of the model into the clinical setting, I propose that machine learning should be used (for excellent reviews on the use of machine learning in medical sciences see Handelman, Rajkomar, Sidey‐Gibbons and colleagues^45^, ^46^, ^47^). Machine learning refers to a broad range of mathematical models used to identify patterns in data, which are predictive of outcome. So here we would build a model that identifies patterns in the vital signs data in order to predict the outcome (either adverse events or pain response depending on our question). The exact choice of model will be dependent on the characteristics of the data and so cannot be specified here. Validation is a key principle of machine learning, providing measures of model performance in independent data so that the generalizability of the model to any given new (never seen before) data can be reliably determined (providing that the test data set is representative of the wider population). Thus, importantly, if we do find a model that is highly predictive of outcome then the model could be translated directly to clinical practice—we do not need to derive a new model for each new infant or hospital, by using machine learning we know how well our model will perform with any new infant. The model should then be converted into software or a clinical device so that it can be easily translated into the clinical setting. Such a device would take an individual infant's vital signs traces and display measures of the predicted outcome, which would be used by clinicians to inform drug regimen or management of the infant. An excellent example of how such a device might work is the HeRO monitor, which uses the infant's vital signs recorded on their standard bedside monitor and calculates measures of heart rate variability to provide an early warning of patient deterioration caused by pathologies such as sepsis, NEC or meningitis.^48^ Moreover, an electronic decision support software has been developed to aid morphine dosing in infants based on pharmacokinetic modelling and the individual's measured morphine concentrations.^27^ This is displayed along with pain scores to inform decisions about the appropriateness of the pain relief. Similarly, a device here could inform clinicians about the effect of an analgesic on vital signs in an individual infant and how this may relate to outcome.

Finally, if this framework proves feasible and suggests that outcome could be predicted from changes in vital signs (and related derived measures), the model would need to be tested in a clinical trial, compared with standard practice, to determine whether its use can improve outcome (Step 4, Figure 1A). For example, if a model predicted the infant's pain response based on changes in the vital signs following analgesic administration and before the painful procedure, this would allow for the analgesic to be titrated accordingly (eg, with an increased dose in those infants who initially did not respond to the analgesic). A clinical trial should be conducted to investigate if the balance of efficacy and adverse effects was improved in a group of infants where analgesic titration was carried out based on the model, compared with a group of control infants who received standard care (ie, the analgesic prescribed without the use of the model). This would be essential to identify whether the model has a benefit in terms of improving health care. Other benefits, such as reduced hospitalization and any cost saving, for example, related to a reduction in adverse events, could also be evaluated. In addition, a clinical trial would allow for any limitations of the model to be assessed. For example, while measurement error (eg, due to poor electrode placement or artifact) should not be a problem when developing a model as it will not occur in all infants, when using the model in an individual infant large amounts of poor data could prove problematic. To alleviate this problem the device should include signal quality metrics, which will highlight to the user periods of poor signal so that probes can be adjusted if necessary and decisions to titrate the drug in question will be informed relative to the quality of the signal.

To understand this framework further let's take some more hypothetical examples (Figure 1B,C). Suppose we average the vital signs from hundreds of individuals (in an exploratory data set – known as a training data set in machine learning), all aligned to the point at which a given analgesic was administered. We find that on average the heart rate drops following drug administration (Figure 1B). So, change in heart rate is a biomarker of drug action, but to determine whether it has use as a biomarker for personalized drug dosing in infants we must investigate whether it varies across infants and whether the change in heart rate can predict outcome. We find that in some infants there is no change in heart rate while in others there is a large drop. When comparing the change in heart rate with measures of efficacy (behavioral, physiological and neurophysiological responses to the painful procedure for which the analgesic was given^23^, ^43^, ^44^) we find that there is a strong relationship, and in this particular case the analgesic does not have any adverse effects. We use machine learning to demonstrate that the rate of change of heart rate shortly after drug administration is predictive of analgesic efficacy. Such a model would suggest that infants with a low initial rate of change in heart rate may benefit from an additional dose of the analgesic. We also build a model, which predicts the time at which the peak drug effect will occur in an individual infant based on the infant's rate of change of heart rate (combining with population pharmacokinetic models), so that the painful procedure can be conducted at the optimal time to receive greatest analgesic benefit for the given infant. We validate these models in an independent test set, and they are converted to a clinical device that informs clinicians whether any given new infant requires an increased dose of the drug to receive analgesic benefit and the optimal time to perform the procedure.

In addition to using vital signs data to identify changes following drug administration, which we have discussed to this point, baseline physiological data (prior to drug administration) can also provide important information. In the schematic example in Figure 1C we see that the adverse effects of the analgesic—an increase in apneas—is related to the number of apneas the infant experiences in the baseline period. While drug adverse effects (and efficacy) will be related to many factors, such as drug absorption and clearance, comedication, duration of administration, and so on, it makes intuitive sense that baseline physiological stability may also have an effect—those infants who are more unstable prior to drug administration may be less resilient to the physiological side effects of a drug leading to an increased risk of adverse effects. Although this is an illustrative example, my colleagues and I recently demonstrated that baseline physiological stability can predict adverse effects in a post hoc analysis^41^ of data collected in the Procedural Pain in Premature Infants clinical trial—a randomized placebo‐controlled trial investigating the safety and efficacy of oral morphine for procedural pain.^49^ We found that the physiological stability of an infant prior to drug administration (measured as number of oxygen desaturations, whether the infant experienced episodes of apnea, average heart rate and average respiratory rate) can accurately predict the infant's risk of adverse cardiorespiratory effects from oral morphine.^41^ The sample size was small as it was a post hoc analysis; nevertheless, this provides an important proof‐of‐principle that machine learning of baseline vital signs could be used to inform provision of pharmacological interventions.

A limitation of the approach outlined here is the assumption that the analgesic (or other pharmacological intervention such as a sedative) has an effect on the vital signs. However, this is likely to be the case for many commonly prescribed analgesics such as opioids.^41^, ^49^ Moreover, these changes will be important to consider for other analgesics as more subtle changes in vital signs may become clear when analyzed across a wide cohort of infants, for example, there is evidence to suggest that paracetamol may cause hypotension^50^ – it is important not to discount changes in vital signs for a particular analgesic before assessing the effect across a large cohort. It is essential to realize though that a different model will need to be developed for every analgesic individually and may also be different depending on factors such as the route of administration of the drug. Additionally, the approach described here is based on the assumption that the side effects of an analgesic (in particular the changes in vital signs) will be related to the analgesic efficacy. If side effects occur through a different mechanism/site of action to the analgesic effect, then this relationship may not necessarily be straightforward. Similarly, the relationship may be disrupted in some individuals who are particularly susceptible to side effects (for example, infants with respiratory distress syndrome may be more susceptible to respiratory depression from opioids). However, machine learning is key to resolving this issue, as nonlinear relationships between side effects and analgesic efficacy can be identified, and using large data sets for both training and testing the model will identify characteristics of infants who follow different patterns, enabling the development of a model that is applicable to all infants.

## 7 A CALL FOR DATA SHARING

How can we achieve these aims? To conduct a machine‐learning analysis, robustly identifying effective biomarkers and validating their relationship with outcome, data from hundreds of infants are ideally needed.^51^, ^52^ Moreover, it is essential to include infants with characteristics representing the range of characteristics across the population as a whole when constructing and validating the model. The rapid developmental changes in factors such as drug absorption, drug metabolism and receptor expression across the neonatal period and into childhood,^6^ as well as the range of pathologies which affect neonates and the variability in infant's baseline vital signs, therefore, accentuates this need for large sets of data. Finally, to fully validate models ensuring they truly can be used in any patient, validation with data sets collected in different hospitals, rather than single center studies, is important.

Given the requirements for large sample sizes and external validation, the best way to advance our understanding of pharmacological effects on infant vital signs and initiate bespoke predictive modelling for personalized drug dosing is to share vital signs data (ie, all available vital signs data obtained direct from infant monitors, including but not necessarily limited to heart rate, oxygen saturation, respiratory rate, and the associated signals from the ECG, photoplethysmograph and impedance pneumograph), along with key demographic and clinical characteristics, such as age and weight of the infant; comorbidities; concomitant medication; type, route of administration, dose and timing of the analgesic; and outcome measures (pain response and adverse events). A concerted collective effort from neonatal clinical and research communities across the globe is needed, but the potential of these data are invaluable and will extend beyond the questions discussed here. Software is freely available to download vital signs data direct from monitors^53^ and with increasing numbers of units centrally monitoring and storing vital signs, along with electronic clinical records, now is the time for this ‘big data’ initiative. Care and attention are needed to ensure that data are shared ethically and the requirements of data protection laws are met (in particular, being fully anonymized), and the data must be shared in a way that is easy to use (for example, by using standardized formats and naming systems, clear labelling of when drugs are given, outcome measures, comorbidities, and standard basic demographic details will be required). A consensus from the neonatal community on these standards should be sort to avoid misunderstanding or time wasted on reorganizing data.^54^ Fortunately other research fields have already developed such initiatives and so there is much guidance in this area,^54^, ^55^, ^56^, ^57^ and some databases of vital signs data already exist such as PhysioNet (which includes very limited numbers of neonatal vital signs, though not related to drug effects).^58^ While this is no easy feat and needs consideration, the benefits clearly out way the costs.

Many factors will affect an infant's vital signs, including (but not limited to) age, sex, comorbidities (such as sepsis and NEC^35^), and mode of ventilation. Moreover, numerous factors affect the pharmacokinetics and pharmacodynamics of a drug, including dose, route, and duration of administration of the drug, comedications, and timing and type of feeding; and there are multiple factors that could affect the infant's response to pain (and so the possible outcome measure of the model) including number and time of previous painful procedures, comfort measures used, and positioning of the infant. Many institutions now store electronic records with much of this information, and so sharing these data alongside vital signs may be relatively straightforward. However, in centers where this is not the case, sharing these data will be time consuming and resource intensive. Nevertheless, many of these variables may not be essential for the model – if we compare vital signs after administration of a drug with baseline vital signs collected before drug administration then many such variables will be accounted for as we are conducting a within subject analysis. Moreover, unknown factors that affect vital signs and transient factors, which are difficult to record, such as sleep state, will also likely be accounted for through analysis of the baseline vital signs. So copious assessment of infant characteristics may not be necessary for every infant and similarly, the occurrence of missing data need not be a problem. Instead, models should be tested against data sets from centers where more detailed demographic information have been shared to identify whether these additional variables improve the model. Furthermore, large data sets, which will more readily be achieved through data sharing, will ensure models are robust to measurement errors (for example, from incorrect placement of electrodes, noise or artifacts on the recordings) and missing data. The increase in power and the timely development of these models, which can only be achieved with large data sets means the benefits of data sharing will likely far outweigh the effects of missing data.

## 8 CONCLUSION

In summary, safe and effective analgesia for procedural pain is urgently required for hospitalized infants. Vital signs are routinely monitored but underutilized in neonatal care and provide the opportunity to investigate differences in physiology, both before and after analgesic administration, which are predictive of individual drug requirements. Through the use of machine learning techniques, we will be able to generate predictive models, and I have outlined a framework to conduct this analysis. To properly validate these models requires large data sets; the best way to achieve this will be through data sharing. This will provide an invaluable resource with which to address this question and many others. Shifting the balance toward efficacy and away from harm through tailored drug regimens is critical for improving care and outcome in hospitalized infants.

## CONFLICT OF INTEREST

I have no competing interests.
